# Supplementary material for: Severe visceral leishmaniasis in Ethiopia: Outcomes, co-infections and mortality in a prospective real-world cohort
Source: PLoS Negl Trop Dis. 2026 Jun 5;20(6):e0013878. doi: 10.1371/journal.pntd.0013878 (PMC13258142; doi:10.1371/journal.pntd.0013878)
Supplement: S1 Text — (DOCX) [file pntd.0013878.s001.docx]

**S1 Text. Additional information on the diagnosis of visceral leishmaniasis**

Tissue aspiration was done for 308 at initial diagnosis. In four patients, it was not done because these were well-known HIV positive patients where after multiple previous VL episodes, parasite clearance could not be achieved anymore and it was decided to start treatment in case of clinical worsening. For two primary VL cases, there were contraindications for splenic aspiration and no bone marrow set was available, hence treatment was started based on a positive rK39 RDT result. All these six cases without tissue aspirate had a positive *Leishmania* blood PCR result at enrolment. Out of 308 undergoing tissue aspiration, 293 were positive. The 15 patients with a negative bone marrow (n=14) or spleen aspirate (n=1) were primary VL cases started on treatment based on a positive rK39 RDT (n=14) or clinical suspicion (n=1). Twelve of these cases were subsequently found to have a positive *Leishmania blood* PCR result.
