# Supplementary material for: Severe visceral leishmaniasis in Ethiopia: Outcomes, co-infections and mortality in a prospective real-world cohort
Source: PLoS Negl Trop Dis. 2026 Jun 5;20(6):e0013878. doi: 10.1371/journal.pntd.0013878 (PMC13258142; doi:10.1371/journal.pntd.0013878)
Supplement: S1 Table — (DOCX) [file pntd.0013878.s002.docx]

**S1 Table. Overview of the main exclusion criteria from the miltefosine/paromomycin trial that were used to define the trial-ineligible group within the study population**^a^

| **Revised (looser) exclusion criteria** |
| --- |
| No parasitological confirmation of VL on tissue aspiration |
| PKDL grade 3 on VL diagnosis |
| Any anti-*Leishmania* drugs < 6 months |
| Age < 4 or > 50 years old |
| VL history (previous VL episode) |
| HIV co-infection |
| Concurrent tuberculosis |
| Female patients of child bearing age not accepting a pregnancy test and/or not agreeing to use contraception |
| Hemoglobin < 5 g/dL |
| Severe VL according to the treating physician, based on clinical manifestations (such as jaundice, bleeding, oedema)^b^ and/or clinically significant laboratory abnormalities^c^ |
| Clinically significant ECG abnormalities at baseline |

VL: visceral leishmaniasis; AmB; PKDL: post-kala azar dermal leishmaniasis

^a^ For Ethiopia specifically, exclusion criteria for malnutrition in adults were based on skin-fold measurements, which were not available in CPS.

^b^ All those with an indication for AmBisome defined by the treating physician based on clinical&laboratory severity indicators were considered trial-ineligible patients; additionally, those with jaundice, oedema, or epistaxis on clinical examination or reporting recent (≤ 7 days) onset epistaxis on admission were considered trial-ineligible patients (even if AmBisome treatment would not be indicated), as was done during the miltefosine/paromomycin trial.

^c^ The following tests were taken into account to define clinically significant laboratory abnormalities: haemoglobin, platelets, liver enzymes, total bilirubin, and creatinine
