# Supplementary material for: Severe visceral leishmaniasis in Ethiopia: Outcomes, co-infections and mortality in a prospective real-world cohort
Source: PLoS Negl Trop Dis. 2026 Jun 5;20(6):e0013878. doi: 10.1371/journal.pntd.0013878 (PMC13258142; doi:10.1371/journal.pntd.0013878)
Supplement: S2 Table — (DOCX) [file pntd.0013878.s003.docx]

**S2 Table. Line list of the main causes of death in VL cases in Gondar, Ethiopia (2023-2024)**

| **Nr** | **Main underlying process or organ affected** |
| --- | --- |
|  | **Severe infection (+/- other conditions)** |
| 1 | Severe hospital acquired pneumonia |
| 2 | Severe hospital acquired pneumonia + septic shock |
| 3 | Severe hospital acquired pneumonia + drug reaction |
| 4 | Severe pneumonia |
| 5 | Severe hospital acquired pneumonia + refractory hypokalemia |
| 6 | Severe hospital acquired pneumonia + exacerbation post-TB bronchiectasis |
| 7 | Septic shock (gastro-intestinal focus) with multi-organ failure (MOF) |
|  | **Severe infection & acute kidney insufficiency (AKI)** |
| 8 | HAP & AKI (drug induced), severe hypokalemia, transfusion-related acute lung injury |
| 9 | Septic shock (gastro-intestinal focus) with multi-organ failure + AKI |
| 10 | Septic shock (gastro-intestinal focus) with multi-organ failure + AKI |
|  | **Severe infection & severe bleeding/coagulation disturbances** |
| 11 | Severe VL + severe HAP + alveolar hemorrhage (decreased platelet/high INR) |
| 12 | Severe sepsis (chest focus) with disseminated intravascular coagulation |
|  | **Severe infection & acute liver failure** |
| 13 | Community acquired pneumonia + septic shock + acute liver failure due to VL |
| 14 | Acute liver failure due to VL/disseminated tuberculosis |
|  | **Acute liver failure** |
| 15 | Acute liver failure due to VL & herbal medication |
| 16 | Acute liver failure due to VL & herbal medication |
| 17 | Acute liver failure due to VL & herbal medication |
| 18 | Acute liver failure due to VL & herbal medication + AKI |
| 19 | Acute liver failure due to VL |
| 20 | Acute liver failure due to VL |
|  | **Other conditions** |
| 21 | Severe epistaxis due to severe trombocytopenia leading to heart failure/severe anemia |
| 22 | Symptomatic hypoglycemia + pulmonary edema |
| 23 | Massive pulmonary embolism |
| 24 | Sudden cardiac arrest (due to massive pulmonary embolism or intracranial bleeding) |

VL: visceral leishmaniasis; TB: tuberculosis; HAP: hospital-acquired pneumonia; AKI: acute kidney insufficiency
