# Supplementary material for: Severe visceral leishmaniasis in Ethiopia: Outcomes, co-infections and mortality in a prospective real-world cohort
Source: PLoS Negl Trop Dis. 2026 Jun 5;20(6):e0013878. doi: 10.1371/journal.pntd.0013878 (PMC13258142; doi:10.1371/journal.pntd.0013878)
Supplement: S3 Table — (DOCX) [file pntd.0013878.s004.docx]

**S3 Table. Prescription of systemic therapeutic antibiotics within 48 hours of VL diagnosis, stratified by CRP values at baseline in VL cases in Gondar, Ethiopia (2023-2024)**

| **CRP values at VL diagnosis**^a^ | **Antibiotics prescribed (< 48 hours of VL diagnosis)** |
| --- | --- |
| <10 mg/L | 13/40 (32.5%) |
| 10-49.9 mg/L | 41/127 (32.3%) |
| 50-99.9 mg/L | 35/86 (40.7%) |
| >100 mg/L | 21/56 (37.5%) |

^a^ As CRP testing was done for study purposes, CRP values were not used by the treating physician when deciding on starting antibiotics

CRP: C-reactive protein; VL: visceral leishmaniasis
